# LysR-type transcriptional regulator OvrB encoded in O island 9 drives enterohemorrhagic *Escherichia coli* O157:H7 virulence

**DOI:** 10.1080/21505594.2019.1661721

**Published:** 2019-09-10

**Authors:** Yutao Liu, Bin Liu, Pan Yang, Ting Wang, Zhanhe Chang, Junyue Wang, Qian Wang, Wendi Li, Jialin Wu, Di Huang, Lingyan Jiang, Bin Yang

**Affiliations:** a TEDA Institute of Biological Sciences and Biotechnology, Nankai University, TEDA, Tianjin, P. R. China; b The Key Laboratory of Molecular Microbiology and Technology, Ministry of Education, Tianjin, P. R. China; c School of Biomedical Engineering, Tianjin Medical University, Tianjin, P. R. China

**Keywords:** Enterohemorrhagic *Escherichia coli*, O157:H7, Genomic island, O islands, locus of enterocyte effacement (LEE), Shiga toxin (Stx)

## Abstract

Enterohemorrhagic *Escherichia coli* (EHEC) O157:H7 (O157) is a major foodborne pathogen that causes severe illness in humans worldwide. The genome of O157 contains 177 genomic islands known as O islands (OIs), including Shiga toxin-converting phages (OI-45 and OI-93) and the locus for enterocyte effacement (LEE) pathogenicity island (OI-148). However, most genes in OIs are uncharacterized and code for unknown functions. In this study, we demonstrated, for the first time, that OI-9 encodes a novel transcriptional activator, Z0346 (named OvrB), which is required for bacterial adherence to host cells and LEE gene expression in O157. OvrB directly binds to the promoter region of LEE1 and activates the transcription of ler (encoding a master regulator of LEE genes), which in turn activates LEE1–5 genes to promote O157 adherence. Furthermore, mouse oral infection assays showed that OvrB promotes O157 colonization in the mouse intestine. Finally, OvrB is shown to be a widespread transcriptional activator of virulence genes in other enterohemorrhagic and enteropathogenic *Escherichia coli* serotypes. Our work significantly expands the understanding of bacterial virulence control and provides new evidence suggesting that horizontally transferred regulator genes mediate LEE gene expression.

## Introduction

Enterohemorrhagic *Escherichia coli* (EHEC) O157:H7 (O157) is an important human gastrointestinal pathogen with the capacity to colonize asymptomatically and cause illnesses ranging from mild watery diarrhea to hemorrhagic colitis and in extreme cases hemolytic uremic syndrome, which is characterized by thrombocytopenia, microangiopathic hemolytic anemia, and acute renal failure [1]. An essential feature of O157 virulence is the ability of cells to form attaching and effacing (AE) lesions on host epithelium that induce the extensive rearrangement of the actin cytoskeleton of epithelial cells, culminating in the formation of pedestal-like structures underneath the bacterial cell [2]. The genes responsible for AE lesions are located within a large pathogenicity island of the bacterial genome known as the locus of enterocyte effacement (LEE), which contains 41 genes grouped into five operons (LEE1–LEE5). LEE1, LEE2, and LEE3 encode the components of the type III secretion system (T3SS) that allows direct injection of bacterial effector proteins into host cells to subvert host cell signaling pathways and AE lesion formation [3]. LEE4 encodes secreted proteins such as *E. coli* secreted protein (Esp)A, EspB, EspD, and EspF [4–6], and LEE5 encodes the adhesion protein intimin and its receptor Tir, which is translocated into the host cell membrane by the T3SS [7]. Shiga toxins (Stx), which are the other main virulence factors, consist of two major types, Stx1 and Stx2 [8]. All EHEC strains produce one or both of the Stx [9], and EHEC O157:H7 strain EDL933 used in this study produces both Stx1 and Stx2 [10].

The complex regulation of LEE expression involves at least three kinds of regulators: LEE-encoded regulators, including Ler (master LEE regulator) [11], GrlA (global regulator of LEE activator), and GrlR (global regulator of LEE repressor) [12]; global regulators, such as H-NS (heat-stable nucleoid-structuring protein), IHF (integration host factor), Fis (factor for inversion stimulation) [13]; and horizontal transferred regulators that include EivF, EtrA (electron transport regulator protein), GrvA (Global Regulator of Virulence A). These regulators drive the transcription of LEE genes either in a Ler-dependent or Ler-independent manner. The Ler-dependent LEE regulators binding directly to the promoter region of LEE1 to activate or repress its transcription, and in turn, control the expression of LEE2 to LEE5 via Ler [11]. In contrast, the Ler-independent LEE regulators exert no regulatory function on the expression of ler and LEE1, while directly regulating one or more other components of the LEE operon. Although LEE mechanisms of T3SS and AE lesion formation have been well characterized, the regulatory network and mechanisms of LEE are still not fully understood.

The genome of EHEC O157:H7 strain EDL933 contains 177 genomic islands known as O islands (OIs) acquired by lateral gene transfer [14]. These OIs contain 1387 genes that account for 26% of the total genes in this strain, with some encoding key virulence factors, such as Shiga toxin and T3SS, in O157 [15]. OI-93 and OI-45 harbor the *Stx1* and *Stx2* genes [15], respectively. OI-148 contains the LEE pathogenicity island. Additionally, genes for non-LEE-encoded effectors are present in OI-122 (*ent*/*espL2, nleB*, and *nleE*) and OI-71 (*nleF, nleH1-2*, and *nleA*) [16,17]; OI-50 and OI-51 harbor genes encoding virulence regulators required for O157 infection [18,19]; OI-48 encoding urease, tellurite resistance (Te^r^), and Iha may contribute to EHEC O157 pathogenesis by promoting adherence of the pathogen [20]. Besides, Z0021 located in OI-1 [21], Z0639 (GmrA) within OI-29 [22], and Z5898 situated in OI-172 [23] are involved in the regulation of bacterial motility and flagellum synthesis. Nevertheless, most genes in OIs have not been characterized and assigned a function yet.

In this study, we identified a novel regulator Z0346, which was named as OvrB (O island-encoded Virulence Regulator B), encoded in OI-9 that activates LEE gene expression to promote adherence, but not motility and flagella biosynthesis in O157. Electrophoretic mobility shift assay (EMSA) and chromatin immunoprecipitation quantitative PCR (ChIP-qPCR) analysis showed that OvrB directly binds to the LEE1 promoter and regulates LEE1–LEE5 expression via the master LEE regulator Ler. We also show that OvrB is a widespread transcriptional activator of virulence genes in different *E. coli* pathotypes among various pathogens. Our results provide insight into the regulatory mechanism of OvrB in O157, as well as a novel example of laterally acquired regulators that tune pathogenicity.

## Materials and methods

### Bacterial strains, plasmids, and cell culture

Bacterial strains and plasmids used in this study are listed in Table S1. Mutant strains were generated using the λ-Red recombination system. Complementary strains were established by cloning *ovrB* into the pACYC-184 plasmid. The strain for OvrB purification was generated by cloning *ovrB* into the pET28a plasmid. The resultant constructs were electroporated into the corresponding strains. Wild-type (WT), mutants, and complementary strains were routinely cultured with shaking at 37°C in Luria-Bertani (LB) broth or agar. When required, isopropyl β-d-thiogalactoside (IPTG) and antibiotics were added to the culture medium at the following final concentrations: 1 mM IPTG, 100 μg/ml ampicillin, 25 μg/ml chloramphenicol, 50 μg/ml kanamycin, 10 μg/ml tetracycline, and 50 μg/ml nalidixic acid. Primers used for all manipulations are listed in Table S2.

### Growth assay

To determine the growth curve of each strain, overnight cultures were diluted 1:1000 in a flask containing 200 ml of LB broth without antibiotics and incubated at 37°C with shaking at 180 rpm. A 100 μl aliquot was removed from the flask and suitable dilutions were plated on LB agar plates. The growth curve was determined by cell counts and is expressed in log_10_ CFU/ml. Experiments were independently performed three times.

### Bacterial adherence assay

Cell adherence assays were performed using HeLa cells and Caco-2 cells, as previously described [24], with some modifications. The cells were purchased from the Shanghai Institute of Biochemistry and Cell Biology of the Chinese Academy of Sciences (Shanghai, China). Before infection, cells were subcultured in a 6-well plate for 12 h and washed three times with pre-warmed phosphate-buffered saline (PBS), and the medium was replaced with fresh DMEM without antibiotics or fetal bovine serum. Cell monolayers were infected with cells in exponential phase (10^8^ colony-forming units [CFU] per well), then incubated for 3 h at 37°C in a 5% CO_2_ atmosphere. Following incubation, cells were washed six times with PBS to remove unattached bacteria, and lysed in 1 ml of 0.1% sodium dodecyl sulfate. Lysate dilutions were plated on LB agar containing appropriate antibiotics, and attachment efficiency was determined by counting the number of bacteria and is expressed in CFU/ml. At least three independent biological replicates were prepared and analyzed.

### Quantitative real-time PCR (qRT-PCR)

RNA samples were isolated using TRIzol LS reagent (Invitrogen; #15596018) and treated with RNase-Free DNase I to eliminate contaminating genomic DNA. cDNA was synthesized using the PrimeScript 1st Strand cDNA Synthesis Kit (Takara; #D6110A), and qRT-PCR was performed using SYBR Green PCR master mix(Applied Biosystems; #4,367,659) on an ABI 7500 sequence detection system (Applied Biosystems, Foster City, CA, USA). The 16S rRNA gene was used as a reference to normalize differences in total RNA quantity among samples. The fold change in target gene relative to *rrsH* was determined by the 2^−ΔΔCt^ method [25]. At least three independent biological replicates were prepared and analyzed.

### Fluorescent actin staining

Fluorescent actin staining (FAS) was performed as previously described [26]. Briefly, overnight bacteria were 1:100 sub-cultured into DMEM and incubated at 37°C with shaking at 180 rpm until an OD600 of 0.6 was obtained, then diluted 1:100 to infect with HeLa cells in the exponential phase on coverslips. After incubation for 3 h at 37°C and 5% CO_2_, the coverslips were washed and fixed with formaldehyde, and the cells were permeabilized with 0.2% Triton-X and stained with fluorescein isothiocyanate-labeled phalloidin to visualize actin filaments. Bacteria and HeLa cell nuclei were stained with propidium iodide. AE lesion formed on each cell were measured; at least 50 HeLa cells per slide, 3 slides each per experiment.

### Motility assay

Overnight cultures were adjusted to an optical density at 600 nm of 1.0, and 1 μl was stab-inoculated using a sterile pipette tip into 0.25% LB agar plates supplemented with appropriate antibiotics as needed. The plates were incubated at 30°C or 37°C for 10 h, and the diameter of the swimming zone around the inoculation site was measured. All strains were tested in triplicate, and the experiment was independently performed three times.

### ChIP-qPCR

ChIP-qPCR was performed as previously described [27].The Δ*ovrB* mutant strain containing an inducible expression vector for 3× FLAG-tagged OvrB was cultured in LB broth at 37°C and 180 rpm until an OD600 of 0.4 and then induced with 1 mM IPTG for 30 min at 37°C, at which point formaldehyde was added to a final concentration of 1% (v/v). After incubated for 25 min, the cross-linking reaction was quenched with glycine to a final concentration of 0.5 M. The cross-linked cells were pelleted and washed three times in ice-cold PBS and sonicated to generate DNA fragments of approximately 300–600 bp. After 12000rpm centrifuge for 20min the supernatant was used as the cell extract for IP of protein-DNA complexes using an anti-3× FLAG antibody (Sigma; #F1804) and protein A magnetic beads (Invitrogen; #10002D) according to the manufacturer’s instructions. An aliquot without the addition of antibody served as the negative control. The protein-DNA complexes were reversed and purified with a PCR purification kit (Qiagen; #28104). To measure the enrichment of potential OvrB-binding targets in the immunoprecipitated DNA samples, relative-abundance qPCR was performed with SYBR green mix. Relative target levels were calculated using the ΔΔCt method [28]. *rpoS* was used as a negative control. The results are reported as the average enrichment for three biological replicates.

### EMSA

EMSA was performed using purified 6× His-tagged OvrB protein expressed in *E. coli* strain BL21 (DE3). LEE1, LEE2-3, LEE4, and LEE5 promoter regions and *rpoS* fragment were amplified by PCR using EHEC O157:H7 strain EDL933 genomic DNA as a template. *Kan* fragment was amplified by PCR using pKD4 DNA as a template. The PCR fragments were purified using SPARKeasy Gel DNA Extraction Kit (Sparkjade; #AE0101-C). Purified PCR fragments (40 ng) were incubated at room temperature for 30 min with various 6×His-tagged OvrB protein concentrations (0–2 µM), in 20 µL reactions containing binding buffer (1 mM Tris-HCl [pH 7.5], 0.2 mM dithiothreitol, 5 mM MgCl2, 10 mM KCl, and 50% glycerol). One micromolar 6× His-tagged OvrB protein was equivalent to 0.684 µg protein in 20 µL reaction system. The protein-DNA fragments were electrophoretically separated on a native polyacrylamide gel at 4°C and 80 V/cm. The gel was stained for 10 min in a solution of 0.1% GelRed (Biotium; #41000), and protein bands were visualized by ultraviolet transillumination.

### Mouse colonization experiments

The virulence assay was performed in mice according to the standards outlined in the Guide for the Care and Use of Laboratory Animals. The experimental protocol was approved by the Institutional Animal Care Committee at Nankai University. All mice were maintained in a specific pathogen-free environment. Before infection, Mice were provided with food and water ad libitum. The mice were orally infected 100 μl PBS containing 10^9^ bacteria growing in logarithmic phase. The infected mice were euthanized by cervical dislocation at 6 h, 2 day, and 4 day post infection. The distal colon was excised, and the luminal contents were removed from each organ. Each distal colon of the intestine was thoroughly and carefully washed, weighed, and homogenized in 0.5 ml of PBS. The homogenates were diluted, and O157 WT strain was plated on LB agar containing 50 μg/ml nalidixic acid; ΔOI-9 mutant, Δ*ovrB* mutant were plated on LB agar containing 25 μg/ml chloramphenicol; and Δ*ovrB* complementary strain was plated on LB agar containing 10 μg/ml tetracycline to determine CFU/g of organ tissue, respectively.

### Statistical analysis

Statistical analysis was conducted using the software MedCalc (v12.3.0.0). The mean ± SD from three independent experiments is shown in figures. Differences between two mean values were evaluated by two-tailed Student’s *t*-test. Statistical significance was assessed using the Mann-Whitney rank-sum test in mouse colonization experiments. A P value < 0.05 was considered to indicate statistical significance.

## Results

### Gene organization and domain structure of OI-9 in O157

**Figure 1.**
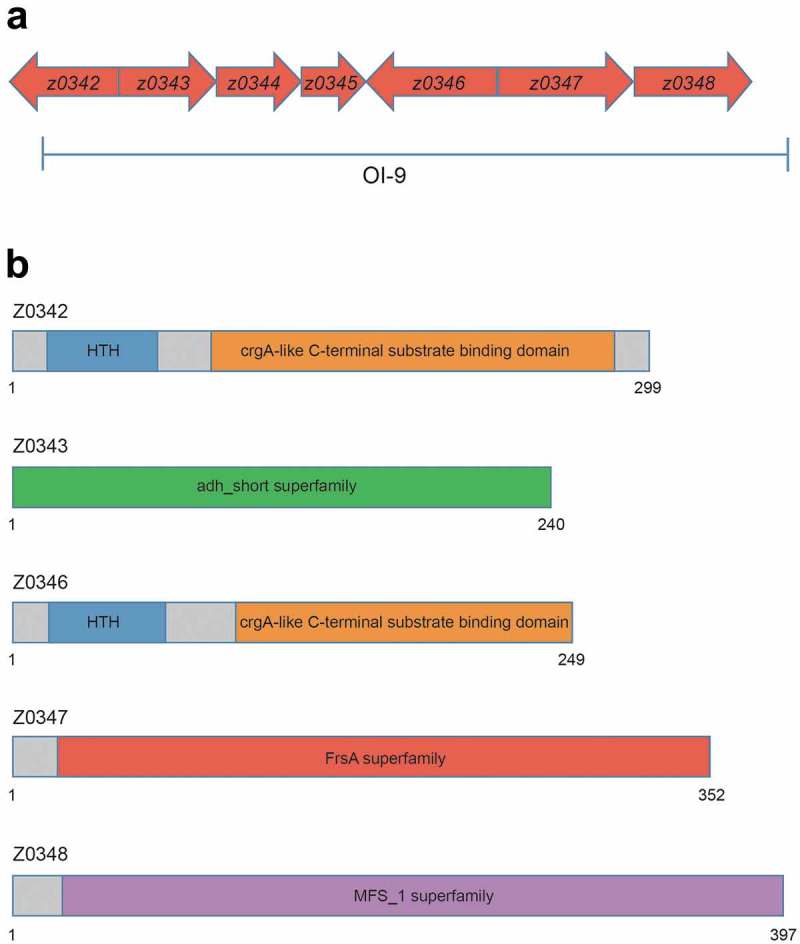
Genetic organization and domain structure of OI-9 in O157. (a) Graphic representation of the region surrounding OI-9 in the genome of O157 EDL933 segment 323657–330128. Arrows represent open reading frames. (b) Domain structure of *z0342, z0343, z0346, z0347*, and *z0348* in OI-9.

OI-9 in O157 is a 6132-bp region containing seven open reading frames from *z0342* to *z0348* (Figure 1(a)). *z0342* and *z0346* (named *ovrB*) encode two putative regulators; while *z0343* encodes an oxidoreductase, *z0347* encodes a hydrolase and the product of *z0348* is a major facilitator superfamily (MFS) transporter. *z0344* and *z0345* encode hypothetical proteins of unknown function. Domain structure analysis revealed that both Z0342 and OvrB contain an N-terminal DNA-binding helix-turn-helix (HTH) motif and a C-terminal co-inducer-binding domain, which are conserved in the LysR-type transcriptional regulator (LTTR) family (Figure 1(b)). LTTRs regulate a variety of virulence determinants and are global regulators of pathogenicity in many bacterial pathogens [29]. Z0343 contains an adh_short superfamily domain, which is the active site of formaldehyde dehydrogenase; thus, Z0343 may be involved in metabolizing endogenous and exogenous aldehydes (Figure 1(b)). Z0344 and Z0345 have no conserved domain, and their functions remain unclear. Z0347 contains a fermentation/respiration switch A domain, which characterizes the DUF1100 family. This family includes several hypothetical bacterial proteins of unknown function (Figure 1(b)). Z0348 contains a MFS_1 superfamily motif that is commonly found in MFS transporters (Figure 1(b)), which were first identified as sugar transporters [30] and were later revealed to have diverse substrates including drugs, Krebs cycle metabolites, organic and inorganic anions, amino acids, lipids, and carbohydrates, among others [31].

### OI-9 is required for O157 adherence and LEE gene expression

To further investigate whether OI-9 is associated with O157 virulence, we first constructed a ΔOI-9 mutant and determined its capacity to adhere to host epithelial cells. ΔOI-9 mutant adhered to HeLa cells at a much lower level, the number of ΔOI-9 mutants adhered to HeLa cells was 2.31-fold less than that of the O157 WT strain (Figure. S1A). A similar level of decrease was also obtained when Caco-2 intestinal epithelial cells were infected instead of HeLa cells (Figure. S1B). O157 WT and ΔOI-9 cells showed similar growth rates, indicating that the difference in adherence capacity between the two strains was not due to differences in cell viability (Figure. S1C). Strikingly, the FAS assay revealed that AE lesion formation on HeLa cells in ΔOI-9 significantly decreased compared with that on O157 WT (Figure. S1D, E). Consistent with the results of the adherence assay and FAS assay, the expression of seven representative LEE genes (*ler, escT, escC, escN, eae, tir*, and *espB*) was significantly downregulated in ΔOI-9 (Figure. S1F). These results suggest that OI-9 is required for O157 adherence to host cells and LEE gene expression.

### OI-9 is not required for O157 motility or flagellar biosynthesis

Flagellum-based motility plays a vital role in bacterial adaptation to environmental conditions and is often associated with pathogen virulence [32]. Therefore, we investigated the effect of OI-9 on O157 motility and flagellar gene expression. The motility assay revealed that O157 WT and the ΔOI-9 mutant showed comparable motility (Figure. S2A), with the radius of the chemotactic ring measuring 1.63 and 1.57 cm, respectively (Figure. S2B). Furthermore, deletion of OI-9 had no apparent effect on the expression of flagellar genes (represented by *flhC, flhD, fliA, fliC*, and *cheY*) (Figure. S2C). These results indicate that OI-9 is not required for O157 motility and flagellar biosynthesis.

### OvrB is a novel LEE regulator in O157

**Figure 2.**
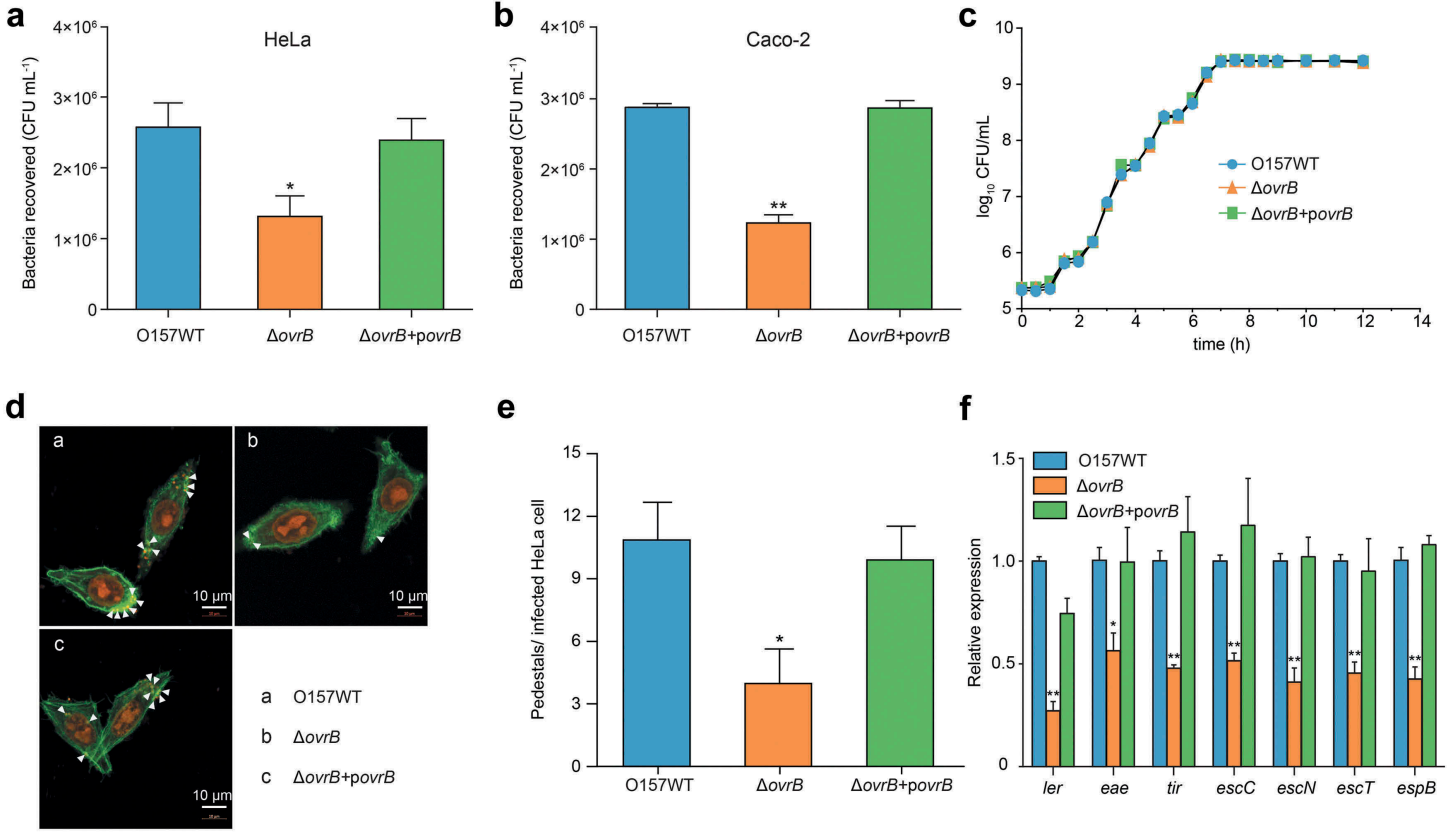
Adherence of Δ*ovrB* mutants *in vitro*. (a,b) Adherence of O157 WT, Δ*ovrB* mutant, and *ovrB* complementary strain to HeLa (a) and Caco-2 (b) cells. (c) Growth of O157 WT, Δ*ovrB* mutant, and *ovrB* complementary strain in LB medium. (d) Detection of AE lesion formation by O157 WT, Δ*ovrB* mutant, and *ovrB* complementary strain by FAS in HeLa cells at 3 h. The HeLa cell actin cytoskeleton (green) and nuclei of bacterial and HeLa cells (red) are shown. AE lesions are indicated by arrowheads. (e) The number of pedestals/infected HeLa cells by O157 WT, Δ*ovrB* mutant, and *ovrB* complementary strain (n = 150 cells). (f) qRT-PCR analysis of changes in LEE gene expression in O157 WT, Δ*ovrB* mutant, and the *ovrB* complementary strain. Data represent mean ± SD (n = 3). *P ≤ 0.05, **P ≤ 0.01, ***P ≤ 0.001 (Student’s t-test).

To further investigate the genes responsible for the defective virulence phenotype observed in ΔOI-9, single deletion mutants (Δ*z0342* to Δ*z0348*) were generated by replacing these genes with a chloromycetin or kanamycin resistance cassette. It was found that the deletion of *ovrB* significantly reduced bacterial adherance to HeLa cells and Caco-2 cells to the same level as that caused by the deletion of the entire OI-9, when compared with O157 WT (Figure 2(a,b)). The growth curve of the Δ*ovrB* mutant was similar to that of the O157 WT strain, demonstrating that the loss of adherence was not due to decreased viability (Figure 2(c)). Consistent with the findings of the adherence assay, FAS showed that Δ*ovrB* mutant formed fewer pedestals on HeLa cells than O157 WT did (Figure 2(d,e)). Accordingly, the transcript levels of LEE genes were downregulated in the Δ*ovrB* mutant relative to O157 WT. All these defects could be rescued by expressing the complementary plasmid pAcyc184-OvrB in Δ*ovrB* mutant (Figure 2(f)). In contrast, deletion of the other OI-9 genes (*z0342, z0343, z0344, z0345, z0347*, and *z0348*) had no visible effect on O157 virulence, since adherence and LEE gene expression levels in all of the mutants were comparable to those of O157 WT (Figs. S3 and S4).

### Activation of LEE genes by OvrB is mediated by Ler

**Figure 3.**
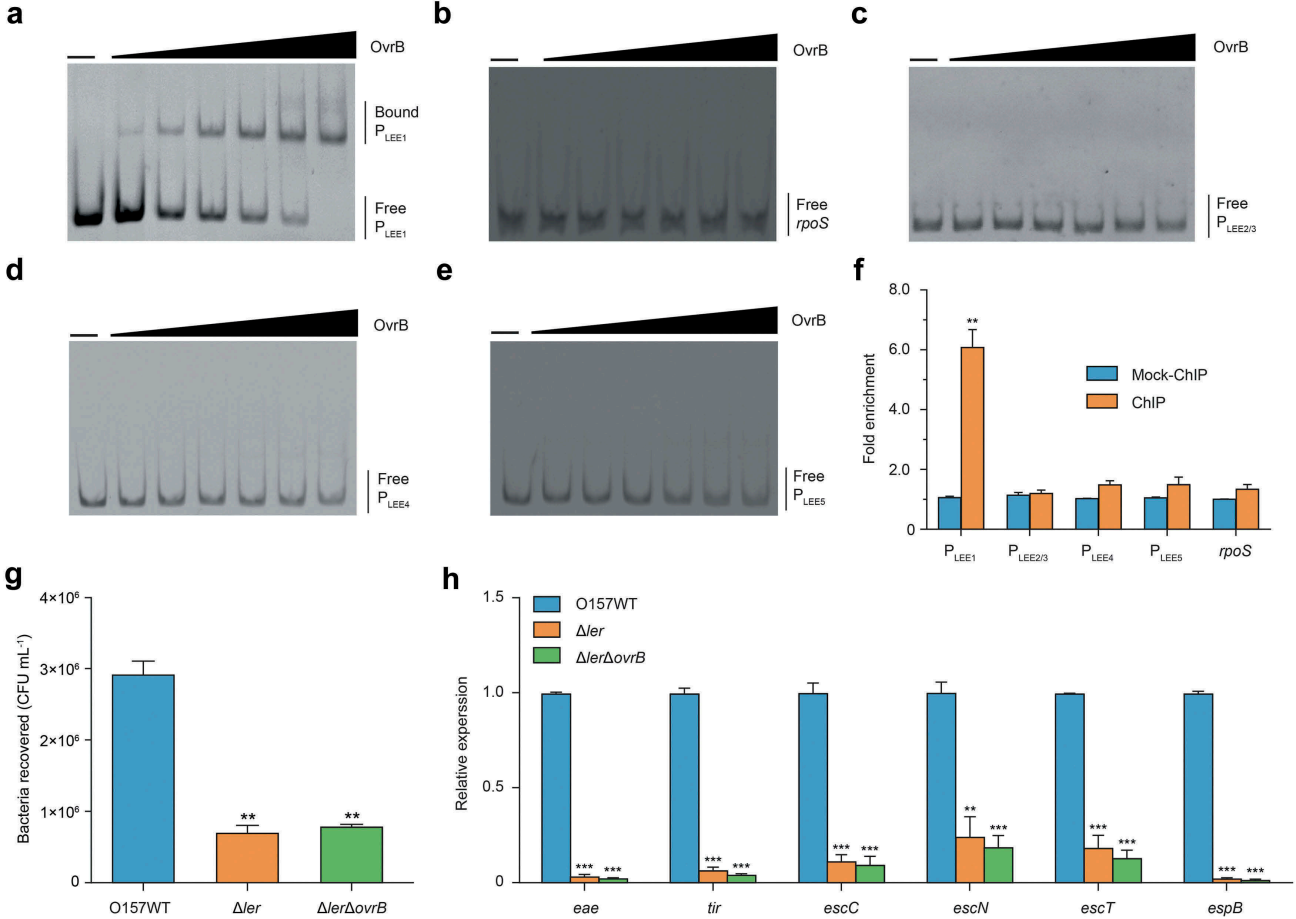
Ler mediates activation of LEE genes by OvrB in O157. (a-e) EMSA of the specific binding of OvrB to P_LEE1_ (a) *rpoS* (negative control) (b), P_LEE2/3_ (c), P_LEE4_ (d), and P_LEE5_ (e). PCR products were added to the reaction mixtures at 40 ng each. OvrB protein was added to the reaction buffer in lanes 2–7 at 0.1, 0.2, 0.4, 0.8, 1, and 2 μM, respectively. No protein was added in lane 1. (f) Fold enrichment of the LEE1, LEE2/3, LEE4, and LEE5 promoters in OvrB ChIP samples, as measured by qPCR. *rpoS* served as negative control. (g) Adherence of O157 WT, Δ*Ler* mutant, and Δ*Ler*Δ*ovrB* double mutant to HeLa cells. (h) qRT-PCR analysis of changes in LEE gene expression in O157, Δ*Ler* mutant, and Δ*Ler*Δ*ovrB* double mutant. Data represent mean ± SD (n = 3). *P ≤ 0.05; **P ≤ 0.01, ***P ≤ 0.001 (Student’s t-test).

To investigate whether OvrB regulates LEE genes directly or indirectly, we first evaluated the binding of OvrB to LEE promoters (P_LEE1_, P_LEE2/3_, P_LEE4,_ and P_LEE5_) by electrophoretic mobility shift assays (EMSA). A mobility shift was observed for P_LEE1_ at increasing concentrations of OvrB (Figure 3(a)), suggesting its direct binding by OvrB. However, there were no noticeable band shifts observed for P_LEE2/3_, P_LEE4_, P_LEE5_, and the two negative controls (*rpoS* and *kan*) under the same conditions (Figure. 3(b-e) and S5). The ChIP-qPCR analysis revealed that P_LEE1_ was enriched 6.07-fold in OvrB-ChIP samples as compared with mock ChIP samples, confirming the binding of OvrB to P_LEE1_ *in vivo*. In contrast, the fold enrichment of P_LEE2/3_, P_LEE4_, P_LEE5_, and *rpoS* did not differ between OvrB-ChIP and mock ChIP samples (Figure 3(f)). These results demonstrate that OvrB directly binds to the promoter region of LEE1 both *in vitro* and *in vivo*.

The first gene of LEE1 encodes the master LEE regulator Ler, which activates the expression of genes from LEE1 to LEE5 [11,33,34]. We next investigated whether the regulation of LEE gene expression by OvrB is Ler-dependent. To this end, we constructed Δ*ler* mutant and Δ*ovrB*Δ*ler* double mutant strains and evaluated their adherence to host cells and expression of LEE genes. Deletion of *ler* in O157 WT markedly reduced both of these properties, which is consistent with the function of Ler as a positive regulator of LEE genes. Furthermore, the adherence capacity and expression of LEE genes in Δ*ovrB*Δ*ler* double mutants were comparable to those in the Δ*ler* single mutant, as determined through the adherence assay and qRT-PCR, respectively (Figure 3(g,h)). These results suggested that the regulatory role of OvrB on O157 adherence and LEE gene expression is mediated by Ler.

### OvrB promotes O157 colonization in vivo

**Figure 4.**
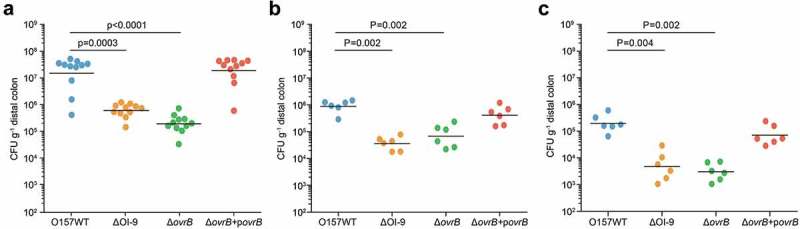
Adherence capacity of O157 in the mouse intestinal tract. (a-c) Evaluation of the adherence capacity of O157 WT, ΔOI-9 mutant, Δ*ovrB* mutant, and Δ*ovrB* complementary strain in distal colon of mice at 6 h (a), 2 d (b), and 4 d (c) post-infection. Horizontal lines represent the geometric means. Statistical significance was assessed with the Mann-Whitney rank-sum test.

We next investigated the role of OI-9 and *ovrB* in O157 colonization *in vivo*. Six-week-old female BALB/c mice were orally infected with O157 WT, ΔOI-9 mutant, Δ*ovrB* mutant, and complementary strain, and the number of bacteria recovered from the distal colon at 6 h, 2 d, and 4 d post-infection were determined to evaluate bacterial colonization capacity. In agreement with the *in vitro* results, fewer ΔOI-9 and Δ*ovrB* mutants were recovered from the distal colon of infected mice compared to that of the O157 WT strain at various infection stages (6 h, 2 d, and 4 d) (Figure 4(a-c)). The number of bacteria recovered was restored to the WT level when a low copy plasmid expressing a functional *ovrB* gene was introduced into the Δ*ovrB* mutant (Figure 4(a-c)). These results demonstrate that OvrB encoded in OI-9 *ovrB* promotes O157 colonization in orally infected mice.

### OvrB is a widespread regulator of virulence in pathogenic bacteria

**Figure 5.**
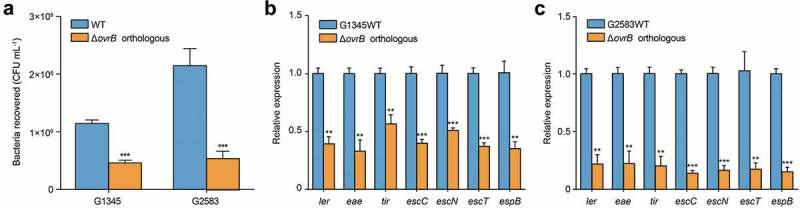
OvrB is a ubiquitous regulator of virulence in pathogenic bacteria. (a) Adherence of G2583 (*E. coli* O55:H7), G1345 (*E. coli* O145:H28), and orthologous Δ*ovrB* mutant to HeLa cells. (b,c) qRT-PCR analysis of changes in LEE gene expression in G2583 (*E. coli* O55:H7) and the orthologous Δ*ovrB* mutant (b) and in G1345 (*E. coli* O145:H28) and the orthologous Δ*ovrB* mutant (c). Data represent mean ± SD (n = 3). *P ≤ 0.05, **P ≤ 0.01, ***P ≤ 0.001 (Student’s t-test).

Bioinformatics analysis of 231 available *E. coli* genome sequences showed that OI-9 and *ovrB* are highly conserved and widely distributed in various *E. coli* lineages. Phylogenetic analysis also revealed that *E. coli* strains with OI-9 are predominantly distributed in two distinct clades consisting entirely of pathogenic strains (Table S3). Clade 1 includes enteropathogenic *E. coli* (EPEC) O55:H7; EHEC strains O157:H7, O145:H28, and O157:H16; and Shiga toxin-producing *E. coli* strain 06–00048 (O36:H14), while Clade 2 comprises extraintestinal pathogenic *E. coli* strains 042, UMN026, PCN033, PPECC42, and ST2747 (Figure. S6). EPEC and EHEC are closely related and share many virulence determinants and features, have similar mechanisms of intestinal colonization through A/E lesion formation and encode a T3SS that translocates multiple effector proteins into the infected enterocyte [35,36]. We investigated whether orthologous *ovrB* genes also regulate adherence capacity by deleting *ovrB* from the representative EPEC and EHEC strains O55:H7 and O145:H28, respectively. The adherence assay results showed that deletion of orthologous *ovrB* significantly reduced bacterial adherence to host cells compared with O157 WT (Figure 5(a)). Consistent with these results, the expression of seven representative LEE genes was also downregulated in these mutants (Figure. 5(b,c)). Thus, OvrB is a ubiquitous LEE activator in different *E. coli* pathotypes.

## Discussion

**Figure 6.**
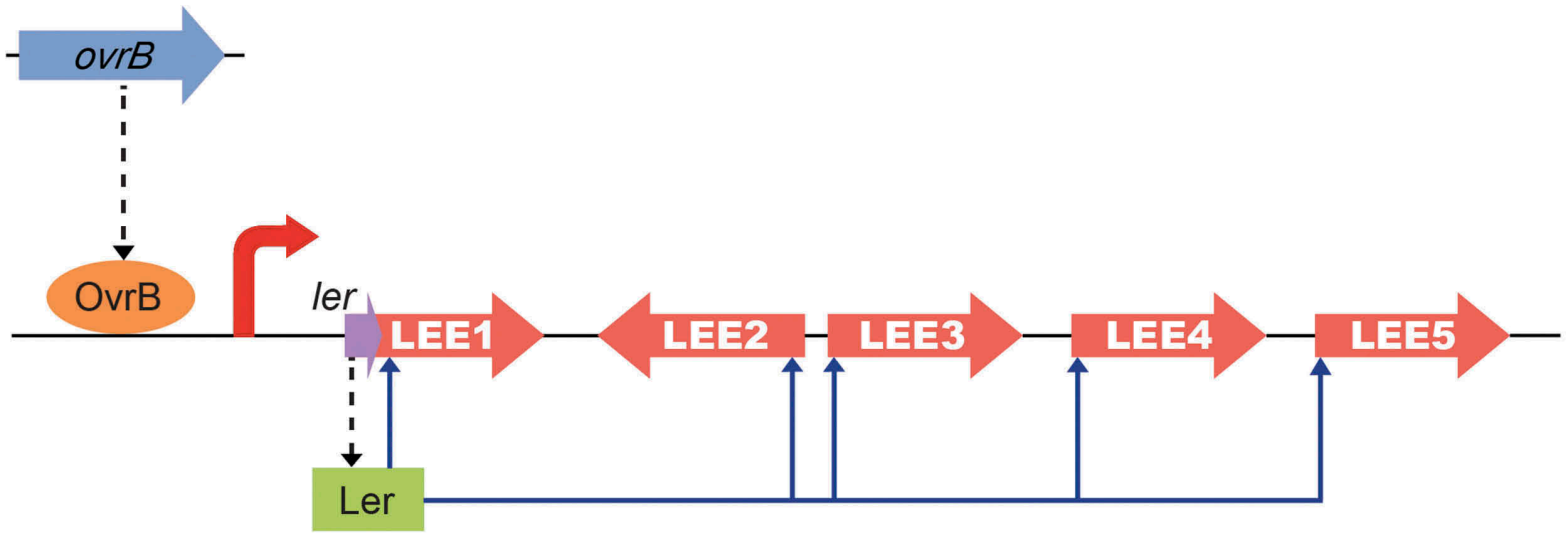
Model of the OvrB regulatory network in O157. Scheme of OvrB regulation of virulence-associated LEE genes. OvrB directly activates LEE1 by binding to the promoter region, which in turn increases the transcript level of *ler*, allowing the Ler protein to function as a downstream activator. Broken black arrows correspond to translated products from these genes. Blue arrows represent input points of positive transcriptional regulation.

O157 is a critical AE pathogen that can cause severe complications in at-risk individuals due to the production of phage-encoded Shiga toxin. The O157 genome contains many OIs, some of which are required for adherence, virulence gene expression, and motility. However, the functions and evolutionary histories of many OIs remain unknown. In this study, we demonstrated that LEE gene levels and adherence capacity were reduced in ΔOI-9 mutants, indicating that OI-9 is involved in O157 virulence and functions by regulating the LEE. OvrB encoded in OI-9 in O157 activates the expression of LEE genes – but not that of flagellar genes – by directly binding to the promoter region of *Ler* and inducing the expression of downstream LEE genes (Figure 6). The transcriptional regulation of LEE genes is complex and controlled by LEE-encoded regulators, global regulators, and horizontal transferred regulators, either in Ler-dependent or Ler-independent manner. These LEE regulators assure LEE expression only under optimal environmental conditions (such as host intestine), while preventing expression to avoid intense metabolic cost and ensuring survival in other environments [37]. Here, we found that OvrB, which is a horizontally transferred regulator, activates LEE expression in an Ler-dependent manner. Our study significantly enhances our understanding about bacterial virulence control by providing a new example of lateral transfer regulators that mediate LEE gene expression; it also increases the complexity of the regulatory network of LEE genes. Undoubtedly, acquisition of more LEE regulators is critical for EHEC O157 to adapt to different changing environments and to evolve into successful human pathogen. However, the other OI-9 genes (*z0342, z0343, z0344, z0345, z0347*, and *z0348*) had no discernible effect on O157 adherence and LEE gene expression. It is proposed that these genes may be redundant or involved in other bacterial processes, which need to be further investigated.

OI-9 is found in different *E. coli* pathotypes, which cluster into two distinct clades. Hence, OI-9 was likely acquired through two independent evolutionary events. For example, the most recent common ancestor of O55:H7, O157:H7, O145:H28, and O157:H16 in Clade 1 appears to have acquired OI-9 after diverging from non-pathogenic *E. coli*. Nearly all strains harboring OI-9 are intestinal pathogens in humans or other animals; these hosts may have driven OI-9 acquisition during the evolution of pathogenic strains. A comparative genomic analysis showed that OI-9 is also present in other reported pathogens, including *Citrobacter freundii, Serratia mareeseens, Enterobacter hormaechei*, and *Klebsiella oxytoca* as well as some other conditioned pathogens (Table S4), suggesting that it is a common feature of pathogenic bacteria and influences pathogenesis. However, the regulation of OI-9 in some LEE-deficient pathogens is yet to be elucidated; it is possible that OI-9 regulates virulence in other ways in these organisms.

OvrB belongs to LysR-type transcriptional regulators that are highly conserved and ubiquitous among bacteria, with functional orthologues identified in archaea and eukaryotes. Members of this family have a conserved N-terminal DNA-binding HTH motif and C-terminal co-inducer-binding domain [38]. LTTRs can act as either transcriptional activators or repressors depending on the location of the HTH domain [39]. LysR-type transcriptional regulators control multiple biological processes including biofilm formation [40] and motility [41] and T3SS [42] in a variety of bacteria including *E. coli, Salmonella enterica* serovar Typhimurium [43], and *Yersinia enterocolitica* [44]. Here, we found the LysR-type transcriptional regulator OvrB promotes O157 adherence by activating LEE gene expression, which significantly expands our insight on the regulatory function and scope of LysR-type transcriptional regulators. Whether OvrB is also involved in the regulation of other biological processes in O157 remains to be established.

## Supplemental material

Supplymentary data for this article can be accessed here.
