# Supplementary material for: LysR-type transcriptional regulator OvrB encoded in O island 9 drives enterohemorrhagic Escherichia coli O157:H7 virulence
Source: Virulence. 2019 Sep 10;10(1):783–92. doi: 10.1080/21505594.2019.1661721 (PMC6768210; doi:10.1080/21505594.2019.1661721)
Supplement: Supplemental Material [file kvir-10-01-1661721-s001.pdf]

LysR-type transcriptional regulator OvrB encoded in O island 9 drives enterohemorrhagic *Escherichia coli* O157:H7 virulence

Yutao Liu[1,2†], Bin Liu[1,2†], Pan Yang[1,2], Ting Wang[1,2], Zhanhe Chang[3], Junyue Wang[1,2], Qian Wang[1,2], Wendi Li[1,2], Jialin Wu[1,2], Di Huang[1,2], Lingyan Jiang[1,2] and Bin Yang[1,2]*

Supplementary Figures

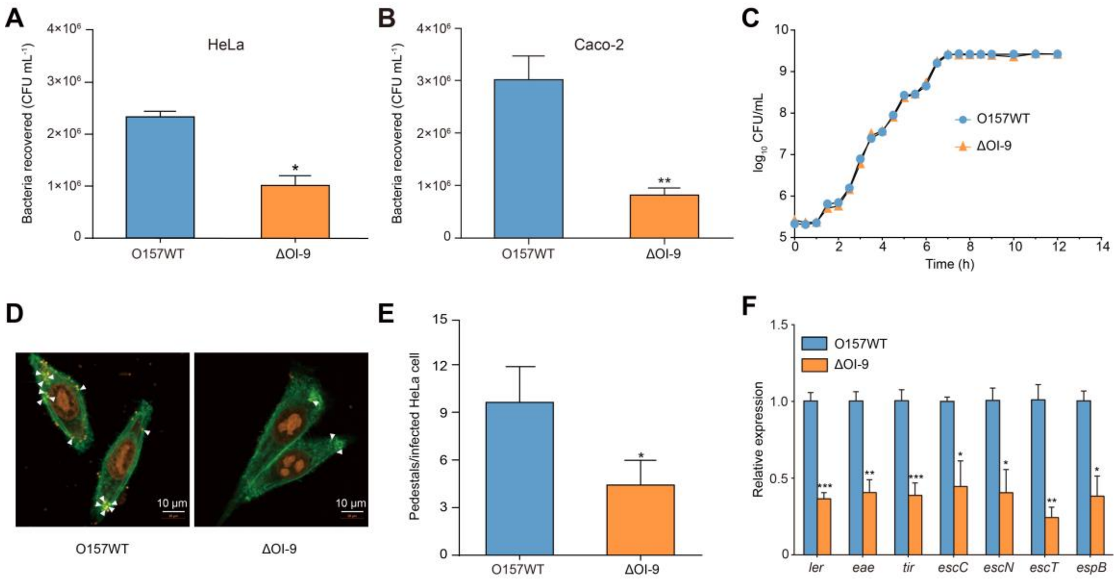

Figure S1. Role of OI-9 in the adherence of O157 in vitro. (A, B) Adherence of O157 WT and the ΔOI-9 mutant to HeLa (A) and Caco-2 (B) cells. (C) Growth of O157 WT and OI-9 mutant in LB medium. (D) AE lesion formation by O157 WT and the ΔOI-9 mutant, as evaluated by FAS in HeLa cells after 3 h. The HeLa cell actin cytoskeleton (green) and nuclei of bacterial and HeLa cells (red) are shown. AE lesions are indicated by arrowheads. (E) The number of pedestals/infected HeLa cell of O157 WT and the ΔOI-9 mutant (n = 150 cells). (F) qRT-PCR analysis of LEE gene expression in O157 WT and the ΔOI-9 mutant. Data represent mean ± SD (n = 3). *P ≤ 0.05, **P ≤ 0.01, ***P ≤ 0.001 (Student's t-test).

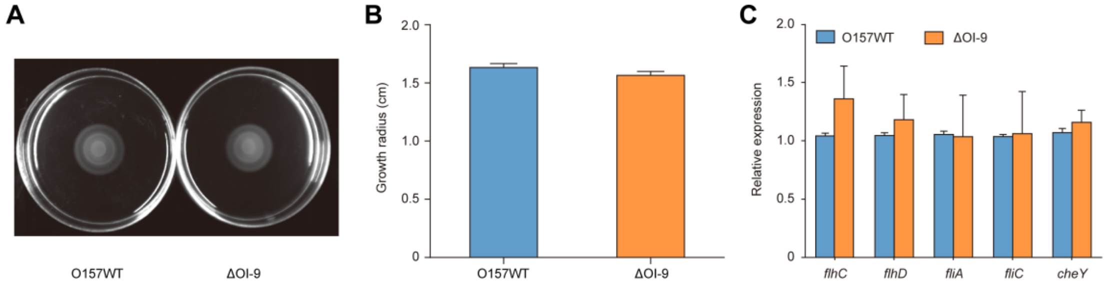

Figure S2. Role of OI-9 in motility and flagellar biosynthesis *in vitro*. (A, B) Representative images of swimming motility (A) and quantification of growth radius (B) of O157 WT and the ΔOI-9 mutant. (C) qRT-PCR analysis of flagellar biosynthesis gene expression in O157 WT and the ΔOI-9 mutant. Data represent mean ± SD (n = 3).

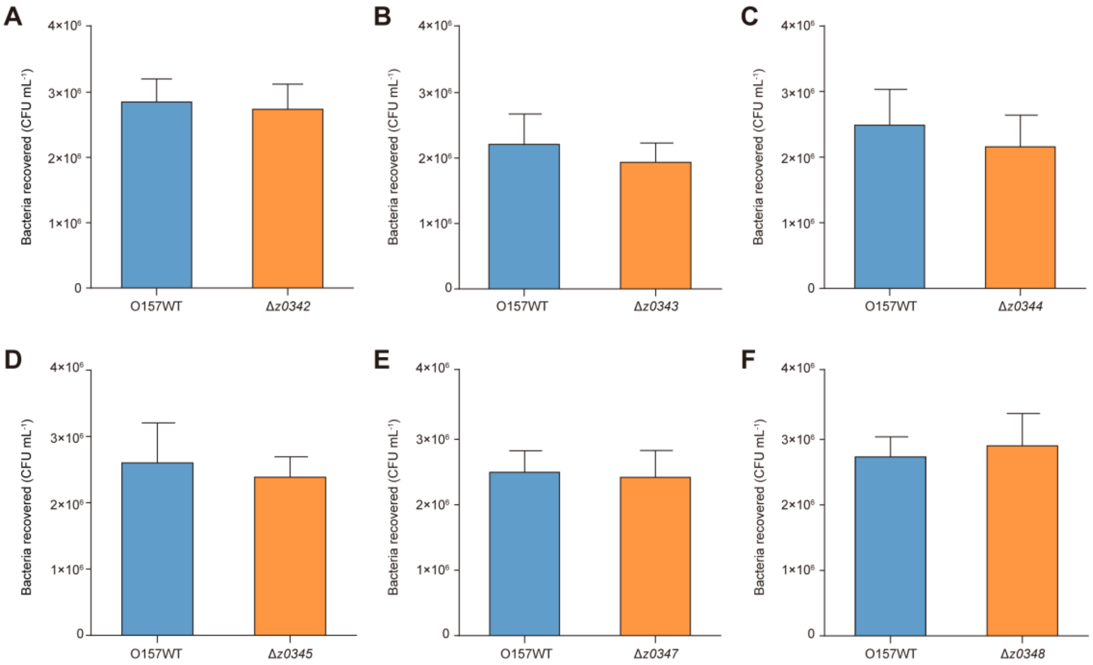

Figure S3. Role of *z0342*, *z0343*, *z0344*, *z0345*, *z0347*, and *z0348* in adherence to HeLa cells. (A–F) Adhesion of O157 WT and Δ*z0342* (A), Δ*z0343* (B), Δ*z0344* (C), Δ*z0345* (D), Δ*z0347* (E), and Δ*z0348* (F) mutants. Data represent mean ± SD (n = 3).

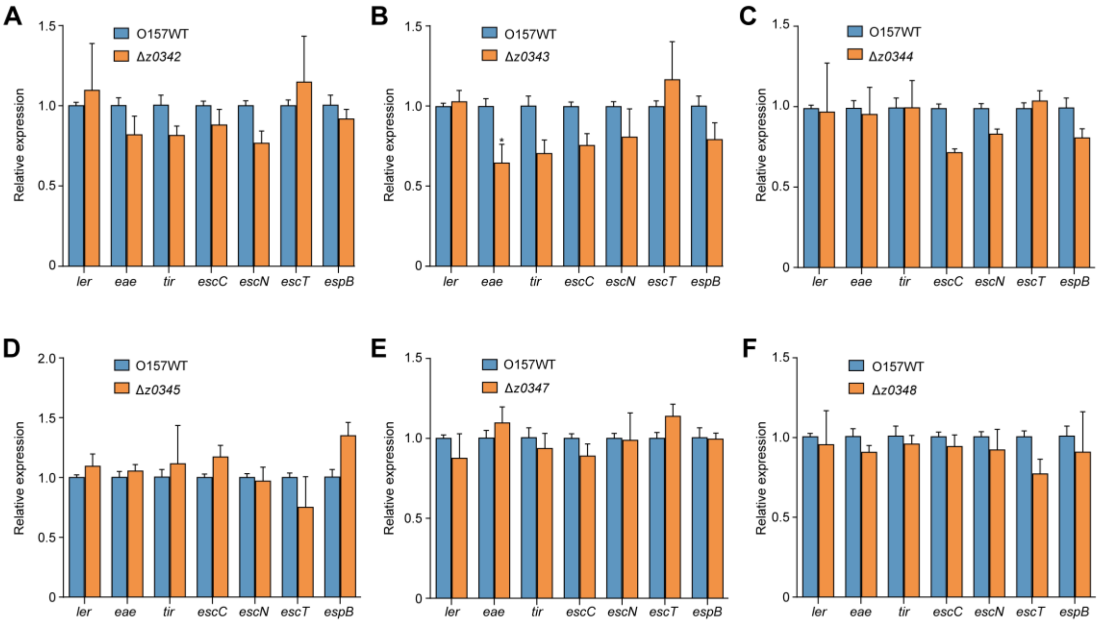

Figure S4. Role of *z0342*, *z0343*, *z0345*, *z0347*, and *z0348* in LEE gene expression. (A–F) qRT-PCR analysis of changes in LEE gene expression in O157 WT and Δ*z0342* (A), Δ*z0343* (B), Δ*z0344* (C), Δ*z0345* (D), Δ*z0347* (E), and Δ*z0348* (F) mutants. Data represent mean ± SD (n = 3).

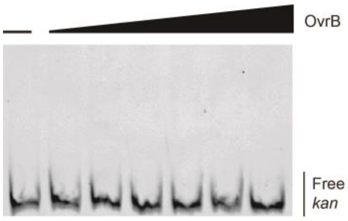

Figure S5. EMSA analysis of binding of the OvrB to the *kan* fragment. PCR products were added to the reaction mixtures at 40 ng each. OvrB protein was added to the reaction buffer in lanes 2–6 at 0.2, 0.4, 0.8, 1, and 2 μM, respectively. No protein was added in lane 1.

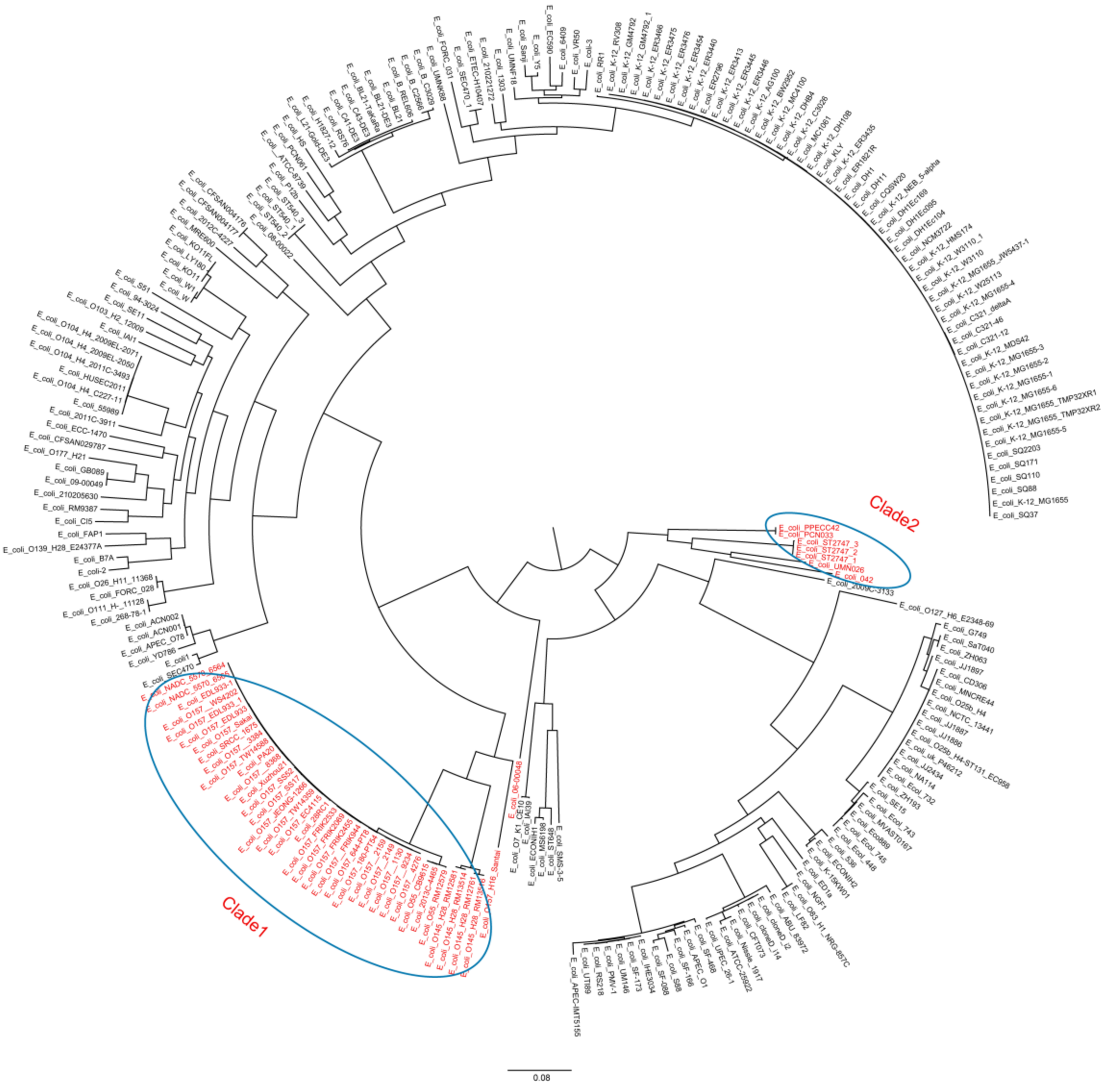

Figure S6. Maximum likelihood tree constructed in PhyML based on 1406 single-copy core genes shared by 231 *E. coli* strains. Strains harboring OI-9 are indicated in red.

Supplementary Tables

Supplementary Table 1. Strains and plasmids used in this study.

**Strains**	**Genotype or description**	**Source or reference**
G2734	Wild-type EHEC O157:H7 EDL933	ATCC*
G1345	Wild-type EPEC O55:H7 CB9615	BfR‡
G2583	Wild-type EPEC O145:H28	BfR‡
H3904	*z0346* orthologues deletion mutant in G1345	This work
H3905	*z0346* orthologues deletion mutant in G2583	This work
H2505	OI-9 deletion mutant in G2734	This work
H2600	*z0342* deletion mutant in G2734	This work
H2577	*z0343* deletion mutant in G2734	This work
H2601	*z0344* deletion mutant in G2734	This work
H2602	*z0345* deletion mutant in G2734	This work
H2578	*z0346* deletion mutant in G2734	This work
H2579	*z0347* deletion mutant in G2734	This work
H2580	*z0348* deletion mutant in G2734	This work
H2674	H2578containing pACYC184-*z0346*	This work
H2663	H2578 containing pTRC99A-*z0346*-3×Flag	This work
H2665	BL21(DE3) containing pET28a-*z0346*	This work
H2661	DH5α containing pACYC184-*z0346*	This work
H2662	DH5α containing pTRC99A-*z0346*-3×Flag	This work
H2664	DH5α containing pET28a-*z0346*	This work
H2165	ler deletion mutant in G2734	This work
H2747	H2165 containing pKD46	This work
H2748	H2578 containing pKD4	This work
H2749	ler deletion mutant in H2578	This work
H2801	G2734 with resistance of nalidixic acid	This work
**Plasmids**		
pKD3	Containing a chloramphenicol resistance cassette and the flipase recognition sites, CmR	BfR
pKD4	Containing a kanamycin resistance cassette and the flipase recognition sites, KmR	Lab collection
pKD46	Red recombination plasmid, ApR	Lab collection
pET28a	T7 expression vector, KmR	Lab collection
pTRC99A	Expression vector, ApR	Lab collection
pACYC184	Expression vector, CmR, TcR	Novagen
pLW1810	pACYC184 carrying *z0346* from G2734，TcR	This work
pLW1812	pET28a carrying *z0346*, KmR	This work
pLW1811	pTRC99A carrying *z0346*-3xFLAG, ApR	This work

[*], ATCC, American Type Culture Collection, Manassas, Virginia, USA. [†], BfR, Beutin Federal institute for Risk Assessment National Reference Laboratory for *Escherichia coli*, Berlin, Germany. [‡], Aus, School of Molecular and Microbial Biosciences, University of Sydney, Sydney, Australia.

Supplementary Table 2. Primers used in this study (5'-3').

Primers for gene mutation		
OI-9	F	GCAACCTCCGGGGACTGACGCCAGCCGATACACTTATGGTGTAGGCTGGAGCTGCTTCG
OI-9	R	GGGCGTGGAAAGCGCTTTCTCACCTCCGGGGGAAATACCCATATGAATATCCTCCTTAG
Z0342	F	AATACTGTTCTGACATCAGGCAATGCAGGTGCAGACATGTGTAGGCTGGAGCTGCTTCG
Z0342	R	CCTGCTTTCAATGTACTCAGTGGTTAGCGGCATTAAACACATATGAATATCCTCCTTAG
Z0343	F	TTCTATTCTCTCTCCGAACACATTTGAAGGAGAACGTAGTGTAGGCTGGAGCTGCTTCG
Z0343	R	TCTGACCTATTTAATAAAACGTGAATGTAAAAGTTATTCCATATGAATATCCTCCTTAG
Z0344	F	GATTATTTTTCACAGGAAGTAAACATGACTCATGTTTAGTGTAGGCTGGAGCTGCTTCG
Z0344	R	GTGCTTCTTGATCATGTCTTAAAAATTATACCATGAGCCCATATGAATATCCTCCTTAG
Z0345	F	TAATGCTGGAGTAATCAATAGACCAGCATTAATCACAGGTGTAGGCTGGAGCTGCTTCG
Z0345	R	CAATACCGTTGTTTTTGTTGCAACAGTAGAATACGCGTACATATGAATATCCTCCTTAG
Z0346	F	ACAATATTGCACAGTGAAGAGTCGGTCGTTGTTAACGTGTGTAGGCTGGAGCTGCTTCG
Z0346	R	ATTAATGCTGAGATTGCCGGCATGAAGGGGAATGTTTCACATATGAATATCCTCCTTAG
Z0347	F	TCATGCTGCTTTTCGCATCATTAAGTGGAGTCACGACAGTGTAGGCTGGAGCTGCTTCG
Z0347	R	CCATAGACCGGTGGCTTTGCATCAATTCAGGGGAGGTTTCATATGAATATCCTCCTTAG
Z0348	F	GCCGGTGGCTTTTATCCTGATCTTTGTGAAATTTCACTGTGTAGGCTGGAGCTGCTTCG
Z0348	R	CTTGTGTAACTTTTTCAGCAACGCGCGCCCTCGCCGGACCATATGAATATCCTCCTTAG
ler	F	TTCCAGCTCAGTTATCGTTATCATTTAATTATTTCATGGTGTAGGCTGGAGCTGCTTCG
ler	R	CTTCCTGATAAGGTCGCTAATAGCTTAAAATATTAAAGCCATATGAATATCCTCCTTAG
z0639-01[*]	F	ACAATATTGCACAGTGAAGAGTCGGTCGTTGTTAACGTGTGTAGGCTGGAGCTGCTTCG
z0639-01	R	ATTAATGCTGAGATTGCCGGCATGAAGGGGAATGTTTCACATATGAATATCCTCCTTAG
z0639-02[†]	F	ACAATATTGCACAGTGAAGAGTCGGTCGTTGTTAACGTGTGTAGGCTGGAGCTGCTTCG
z0639-02	R	ATTAATGCTGAGATTGCCGGCATGAAGGGGAATGTTTCACATATGAATATCCTCCTTAG
Primers for identifying the gene mutants		
OI-9	F	CAAGAGCCAGCCTCAACATC
OI-9	R	CGATACGGCGGTCATTTTG
z0342	F	TGGTGGCGATTATTGGTTCC
z0342	R	CTCTGCGTGCTCCCAGTAAAA
z0343	F	CAGGATACTGGAACCAATCATAAAG
z0343	R	AGAGGAAGCCCCACTCGTTAT
z0344	F	ATTATTGAATCTCCCGAAAGCG
z0344	R	CTCTACCACTCCAGACCGAAGC
z0345	F	TGCTTCGGTCTGGAGTGGTAG
z0345	R	ACAAGGGGACTATCCAGCAATG
z0346	F	GCACAGTGAAGAGTCGGTCGT
z0346	R	GTCCCCGTAATCCTAACCAACT
z0347	F	GACCCCATCAAAGTAAAGCACC
z0347	R	CCACTACCACAAACCGAATCAC
z0348	F	GGTTGGCAGTAAAGACAAAGAGTT
z0348	R	GGGTATCACCAACGAGTAGCG

kan	F	GCCGATTGTCTGTTGTGCCC
kan	R	CGGTGCCCTGAATGAACTGC
chl	F	GGAGTGAATACCACGACGAT
chl	R	ATTGGCTGAGACGAAAAACA
ler	F	TTATTTCTTGTTTGGCTCAC
ler	R	ATTGTTGGTCCTTCCTGAT
z0639-01[#]		ACAATATTGCACAGTGAAGAGTC
z0639-01		ATTAATGCTGAGATTGCCGGCA
z0639-02[$]		AGTGAAGAGTCGGTCGTTGTTA
z0639-02		ACAGTGAAGAGTCGGTCGTTGTT
Primers for pKD46 identifying		
pKD46	F	TGAAATGCCCGTTTACCT
pKD46	R	GCCCGACTGATACGTTGA
Primers for G2734 identifying		
WZX	F	CTTGGTGCTGCTCTGACATT
WZX	R	AATCGTAAGAGGGACCGTAGA
fliC	F	TCACAGTTGGCGGCGTAG
fliC	R	CATCAGCATGAAGGGTGGC
Primers for gene cloning		
z0346	F	CATGCCATGGCAAAAAGGGAGAACTACAAC
z0346	R	CGCGGATCC TCAGATTTTCAATGTATCAATGAC
Primers for *Z0346*-3×FLAG		
z0346	F	CGGGAATTCATGGCAAAAAGGGAGAACTACAAC
z0346	R	CGCGGATCCTTACTATTTATCGTCGTCATCTTTGTAGTCGATATCATGATCTTTATAATCA CCGTCATGGTCTTTGTAGTCGATTTTCAATGTATCAATGAC
Primers for CHIP-qPCR		
P_{LEE1}	F	GCTTGGTTTTTATTCTGTTTTATTTGT
P_{LEE1}	R	ACATCTATTTCATCAAACAACCACC
$P_{LEE2/3}$	F	ATTTCCCAATAATCTTAAAAACTCTTC
$P_{LEE2/3}$	R	AATCACTCCTGTCTTCTCATCCAC
P_{LEE4}	F	CCGATTACGCATCGCACC
P_{LEE4}	R	CATTAGCCATTGGAAACTCACG
P_{LEE5}	F	ATGTTCAGCCGTTTATCGACTAC
P_{LEE5}	R	GGGATTATGACCAAGATTACCAA
rpos	F	GTTATCGCAGGGAGCCACA
rpos	R	TTTTACCACCAGACGCAAGTTA
Primers for EMSA		
P_{LEE1}	F	TCCTGGGGATTCACTCGCTTG
P_{LEE1}	R	TCATAATAAATAATCTCCGC
$P_{LEE2/3}$	F	CATTACTGCACCAGAAGGAC
$P_{LEE2/3}$	R	AGATTCATCTGCAGGCTCTG
P_{LEE4}	F	CGCATCGCACCATTGAGAAG

P_{LEE4}	R	CATTAGCCATTGGAAACTCACG
P_{LEE5}	F	TAGTTTGCTTAATTGGTTTTCTTTGGC
P_{LEE5}	R	ACCAATAGGCATAAATATCTC
rpos	F	GTTATCGCAGGGAGCCACA
rpos	R	TTTTACCACCAGACGCAAGTTA
kan	F	TCACTCATTAGGCACCCCAG
kan	R	CTCTTCGCTATTACGCCAGC
Primers for RT-PCR		
rrsH	F	GAAAGCGTGGGGAGCAAAC
rrsH	R	ACATGCTCCACCGCTTGTG
escC	F	GACCAAAATGTTGTCGTCCCA
escC	R	AGGTTACCGCTTCGCTCG
eae	F	GACGGTAGTTCACTGGACTTCTT
eae	R	TCGCCACCAATACCTAAACG
tir	F	AAAGCAGCAGGCGAAGAGG
tir	R	TCGGCACCTGCGAATCAT
ler	F	CAGGAAGCAAAGCGACTG
ler	R	ACCAGGTCTGCCCTTCTT
escT	F	GCAATAGATGCGGCTGGAC
escT	R	TCGGCTTGTAATGGTAATATCTCG
escN	F	AGGTTTTCTTGTTGCCTTTTGA
escN	R	TCTCCATTGGTCTGCCTATGC
espB	F	AAAACTCCTCGGCAAGATGG
espB	R	AATAATCCCGCCAACCAAAG
flhC	F	GTAATGGCGTCGATGCGGTGAT
flhC	R	CCAATGTCCAGGCACGGGTTAA
flhD	F	CGTTAGCGGCACTGACTCTTCC
flhD	R	TCAGCAAGCGTGTTGAGAGCAT
fliA	F	GATGAACTTCGCAGCCGTGACT
fliA	R	GATGAACTTCGCAGCCGTGACT
fliC	F	TCACAGTTGGCGGCGTAG
fliC	R	CATCAGCATGAAGGGTGGC
cheY	F	ATGTTGAGGAAGCGGAAGATG
cheY	R	AGTCACCATTAACACTGGCAAT
z0342	F	GCGTAAGGCTCGGAGAGGTCAT
z0342	R	TGCTGTTGGCTGCGAGATAGGA
z0343	F	AATCCGTGTGACCTGCGTAAATCC
z0343	R	CAGAACGGTAGGCGTCCATTGC
z0344	F	ACTCATCCTCTCAATTGTCAGGGAA
z0344	R	ATGGTTGAACCGACGGCAGTAAT
z0345	F	CAGACAGAAATGGTTAAATCGTCGA
z0345	R	CGGCAATAAACTGTTCATTTCACTG

z0346	F	GGGTCTTGCTCAGTCAGGAGTCA
z0346	R	CTGCTTGCGTCAGCGACAGTT
z0347	F	GGATGGCGAAGACCGTTATTCTACC
z0347	R	CACTGACCTTGCCGTTGCTATCG
z0348	F	TCTGGATGGTCTCGCTGCTGTT
z0348	R	GCTTCGGCGTTGTCAGGCAA

*, [#], *z0346*-01, *z0346* orthologues in G2583; †,[$] *z0346*-02, *z0346* orthologues in G1345;

Supplementary Table 3. The orthologous genes of *ovrB* in different sequenced *E. coli* genomes

				Identical with *ovrB* (%)	
Strains	Pathotype*	Clade	Orthologous gene	Nucleic acid	Amino acid
Escherichia coli O157:H7 str. Sakai	EHEC	Clade1	ECs_0309	100	100
Escherichia coli O157:H7 str. TW14359	EHEC	Clade1	ECSP_RS01640	100	100
Escherichia coli O157:H7 str. Xuzhou21	EHEC	Clade1	CDCO157_RS01620	100	100
Escherichia coli O157:H7 strain FRIK2069	EHEC	Clade1	A8V30_01765	100	100
Escherichia coli O157:H7 strain FRIK2455	EHEC	Clade1	A8V32_01770	100	100
Escherichia coli O157:H7 strain FRIK2533	EHEC	Clade1	A8V31_01765	100	100
Escherichia coli O157:H7strain SRCC 1675	EHEC	Clade1	AR439_19665	100	100
Escherichia coli O157:H7 strain 28RC1	EHEC	Clade1	ARC77_20930	100	100
Escherichia coli O157:H7 strain JEONG-1266	EHEC	Clade1	JEONG1266_18765	100	100
Escherichia coli O157:H7 strain WS4202	EHEC	Clade1	AO055_01605	100	100
Escherichia coli O157:H7 str. SS52	EHEC	Clade1	SS52_RS01620	100	100
Escherichia coli O157:H7 str. SS17	EHEC	Clade1	SS17_RS01620	100	100
Escherichia coli O157:H7 str. EC4115	EHEC	Clade1	ECH74115_RS02170	100	100
Escherichia coli O55:H7 str. RM12579	EPEC	Clade1	ECO55CA74_RS01875	99.89	99.66
Escherichia coli O55:H7 str. CB9615	EPEC	Clade1	G2583_RS01880	99.89	99.66
Escherichia coli strain 2013C-4465	STEC	Clade1	CSR56_18075	99.89	99.66
Escherichia coli O145:H28 str. RM12581	EHEC	Clade1	ECRM12581_RS01295	98.34	98.64
Escherichia coli O145:H28 str. RM13514	EHEC	Clade1	ECRM13514_0260	98.34	98.64
Escherichia coli O145:H28	EHEC	Clade1	ECRM12761_RS01300	98.34	98.64

str. RM12761					
Escherichia coli O145:H28 str. RM13516	EHEC	Clade1	ECRM13516_0258	98.34	98.64
Escherichia coli 06-00048	STEC	Clade1	GJ11_01660	98.19	98.30
Escherichia coli ST2747	ExPEC	Clade2	CF60_05715	97.97	98.30
Escherichia coli UMN026	ExPEC	Clade2	ECUMN_0311	97.97	98.30
Escherichia coli PCN033	ExPEC	Clade2	PPECC33_RS01625	97.85	97.96
Escherichia coli O157:H16 str. Santai	EHEC	Clade1	CP007592	97.85	97.96
Escherichia coli 042	ExPEC	Clade2	EC042_RS01660	97.74	97.96
Escherichia coli PPECC42	ExPEC	Clade2	AWH70_RS01620	97.74	97.96

*: EHEC, enterohemorrhagic *Escherichia coli*; EPEC, enteropathogenic *Escherichia coli*; STEC, Shiga toxin-producing *Escherichia coli*; ExPEC, extraintestinal pathogenic *Escherichia coli*;

Supplementary Table 4. The blast results of OI-9 in different representative intestinal pathogens

Strains	Query cover	Identity
Enteropathogenic *Escherichia coli* O55:H7	100%	99%
Citrobacter freundii strain BD	61%	93%
Serratia marcescens subsp. marcescens Db11	57%	88%
Enterobacter cloacae strain GEO_49_Up_B	54%	75%
Klebsiella oxytoca strain CAV1374	55%	80%
